# Cardiorenal Syndrome Type 1 Complicated by Uremic Pericarditis and a Small Pericardial Effusion Causing Cardiac Tamponade

**DOI:** 10.7759/cureus.107573

**Published:** 2026-04-23

**Authors:** Fadi Khoury, Dhruva Govil, Kelli Kosako-Yost, Kevin Lin, Clement Singarajah

**Affiliations:** 1 Cardiology, The University of Arizona College of Medicine, Phoenix, USA; 2 Internal Medicine, Henry Ford Providence Southfield Hospital, Southfield, USA; 3 Gastroenterology, The University of Arizona College of Medicine, Phoenix, USA; 4 Critical Care Medicine, The University of Arizona College of Medicine, Phoenix, USA

**Keywords:** cardiac tamponade, cardiorenal syndrome, heart failure, pericarditis, uremia

## Abstract

Cardiac tamponade is typically associated with large pericardial effusions; however, even small effusions can result in significant hemodynamic compromise, particularly in the setting of rapid fluid accumulation and underlying cardiac pathology. This case highlights the diagnostic challenge of identifying low-volume tamponade in a patient with elevated baseline intracardiac pressures. An elderly man was admitted for acute heart failure complicated by cardiorenal syndrome. Following initial diuresis, he developed paradoxically worsening hypotension and pleuritic pain. The absence of a pericardiocentesis, along with clinical recovery, suggested that the patient experienced a spectrum of pericardial constraint rather than definitive, high-pressure tamponade. Severe uremia and a small pericardial effusion suggested uremic pericarditis. Swan-Ganz catheterization showed elevated and relatively concordant diastolic pressures. While diuresis initially complicated the clinical picture, anti-inflammatory therapy with prednisone and colchicine resulted in clinical improvement. This report serves to define the importance of multimodal hemodynamic assessment when classic echocardiographic signs of tamponade are masked by preexisting cardiac remodeling.

## Introduction

Cardiac tamponade typically occurs with large pericardial effusions; however, even small effusions can cause hemodynamic compromise, particularly in the setting of rapid fluid accumulation and underlying cardiac pathology. The hemodynamic impact of a pericardial effusion depends on the rate of accumulation and pericardial compliance. In patients with chronic heart failure (HF), the pericardium may be less compliant, meaning that even small increases in fluid can cause a precipitous rise in intrapericardial pressure that exceeds intracardiac diastolic pressure. Standard markers, such as right-sided collapse, can be frequently absent if baseline intracardiac pressures remain high enough to resist extrinsic compression [1].

Uremic pericarditis is a rare but serious complication of advanced kidney dysfunction, which can progress to tamponade in nondialyzed patients. Although echocardiography is the primary screening tool, early presentations are sometimes atypical. This case demonstrates that in complex patients, "pericardial constraint," a state where the pericardium limits cardiac filling, may be a more accurate physiological description than classic cardiac tamponade.

## Case presentation

A 75-year-old man presented with a history of paroxysmal atrial fibrillation on apixaban, coronary artery disease status post percutaneous coronary intervention (left anterior descending and right coronary artery), CKD G3a A2, hypertension, and non-insulin-dependent diabetes presented with subacute, progressive dyspnea and bilateral lower extremity edema. Upon presentation, he was hypertensive, normocardic, and saturating 88% on room air. Initial physical exam revealed bibasilar fine crackles and 3+ bilateral lower extremity pitting edema.

**Figure 1:**
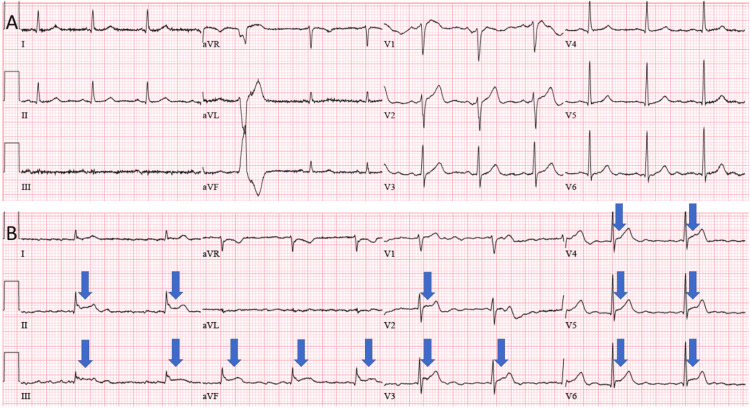
ECGs during hospitalization. (A) ECG on admission showing normal sinus rhythm with premature ventricular complex and lack of low voltage or electrical alternans. (B) ECG obtained on upgrade to the ICU showing diffuse ST segment elevation (highlighted by blue arrow), though still lacking low voltage or electrical alternans aVR: augmented vector right; aVL: augmented vector left; aVF: augmented vector foot

Initial chest X-ray revealed pulmonary vascular congestion, and ECG showed normal sinus rhythm with premature ventricular complex (Figure 1). Initial creatinine was 1.37 mg/dL from a baseline of 1.15 mg/dL (reference range, 0.6-1.2 mg/dL), and blood urea nitrogen (BUN) was 24 mg/dL (reference range, 6-20 mg/dL), with an elevated B-type natriuretic peptide and negative troponin. He was admitted for the management of acute-on-chronic HF.

**Figure 2:**
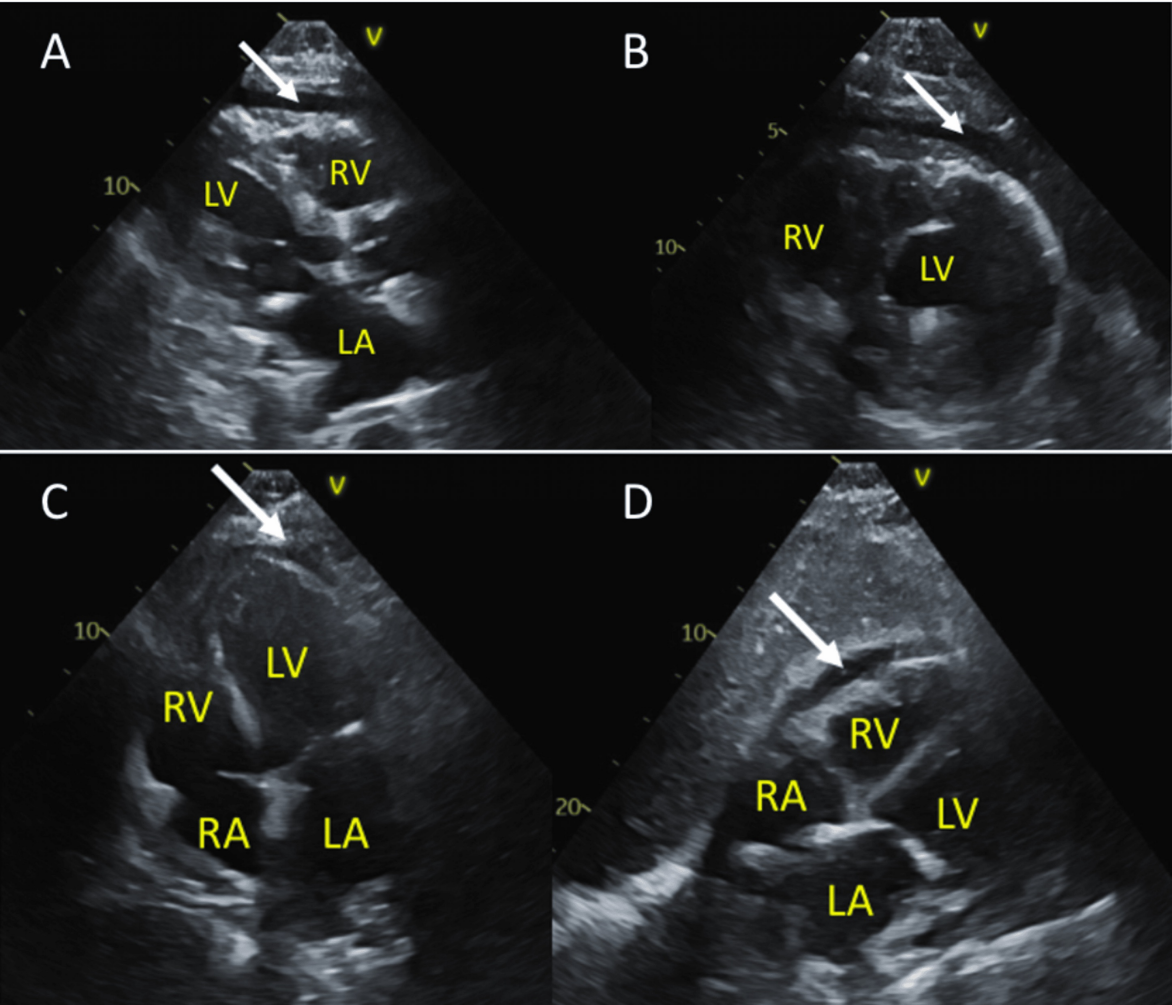
Pericardial effusion on transthoracic echocardiogram. Small pericardial effusion (<1 cm in diameter) is indicated by white arrows. Despite its small volume, the rapid accumulation of fluid resulted in obstructive physiology. Also note the absence of right ventricular collapse. (A) Parasternal long-axis view. (B) Parasternal short-axis view. (C) Apical four-chamber view. (D) Subxiphoid view LA: left atrium; LV: left ventricle; RA: right atrium; RV: right ventricle

On day 2, transthoracic Doppler echocardiography (TTE) showed moderate diastolic dysfunction and elevated right ventricular systolic pressure of 44 mmHg. By day 4, he developed hypotension (BP 81/46 mmHg, mean arterial pressure (MAP) 58 mmHg) and distant heart sounds. Laboratory evaluation revealed a serum lactate of 2.4 mmol/L and an arterial blood gas showing a pH of 7.32, consistent with early tissue hypoperfusion despite a preserved cardiac index. Furthermore, creatinine was 2.06 mg/dL, and BUN was 80 mg/dL (peaking at 5.04 and 132 mg/dL, respectively), consistent with severe azotemia. Initial management included IV furosemide for suspected cardiogenic shock; however, this resulted in immediate hemodynamic collapse. This paradoxical response to volume removal shifted the working diagnosis toward obstructive physiology. Once in the ICU, bilevel positive airway pressure was started for dyspnea relief, and a Swan-Ganz catheter was placed for further hemodynamic assessment (Tables 1, 2). Given the worsening renal function, near-equalization of diastolic pressures, and refractory hypotension, a multidisciplinary evaluation was done. Nephrology consultation for possible hemodialysis (HD) was deferred due to hypotension and the facility’s lack of continuous renal replacement therapy (CRRT). With an MAP of <60 mmHg, norepinephrine was initiated, and anticoagulation was held out of concern for hemorrhagic effusion. Anti-inflammatory therapy with prednisone and renally dosed colchicine was initiated. The decision to prioritize anti-inflammatory therapy over mechanical drainage was based on the high procedural risk and small effusion size (diameter <1 cm) (Figure 2).

**Table 1:** Swan-Ganz catheter hemodynamic data SvO_2_: venous oxygen saturation

Parameter	Pressure (mmHg)	Reference range (mmHg)	SvO_2_ (%)	Reference range (%)
Right atrium	26/22	<10	53	70-75
Right ventricle	38/26	15-30/0-8	52	70-75
Pulmonary artery	44/23	15-30/5-16	52	70-75
Pulmonary artery occlusion pressure	25	6-12	52	70-75
Pulmonary capillary wedge pressure	25	4-12	56	70-75

**Table 2:** Swan-Ganz catheterization data Data showing hemoglobin, central venous pressure, and Fick's calculations for cardiac output and cardiac index. This table shows the near equalization of diastolic pressures, a hallmark of pericardial constraint. While the cardiac index is preserved, these elevated, equalized pressures and a low PAPi substantiate a diagnosis of pericardial constraint rather than isolated hypervolemia PAPi: pulmonary artery pulsatility index

Parameter	Values	Reference range
Hemoglobin (g/dL)	10.4	13.5-17.5
Central venous pressure (mmHg)	24.0	<10
Cardiac output (Fick; L/min)	6.0	4-8
Cardiac index (Fick; L/min/m^2^)	2.6	2.5-4.2
PAPi	0.88	>1.5-2.0
Systemic vascular resistance (Wood unit)	5.7	10-15

After four days of therapy, his hemodynamic status improved, and he returned to the general floors. This clinical recovery without mechanical intervention suggests that the reduction in pericardial inflammation improved diastolic compliance. The patient’s pericarditis therapy was continued for the remainder of the hospitalization with resolution of symptoms and return to baseline kidney function. He was discharged on a six-week course of prednisone and colchicine. Eight days later, he was readmitted with recurrent HF and cardiorenal syndrome. Repeat TTE showed persistent pericardial effusion, now moderate in size (1.3 cm), yet notably without the previous hemodynamic compromise, suggesting a transition to a more chronic, compliant effusion. Symptoms improved with gentle diuresis and continued pericarditis treatment. Upon outpatient follow-up, the patient reported complete symptom resolution.

## Discussion

Cardiorenal syndrome type 1 (CRS1) involves acute HF precipitating acute renal failure. Reduced perfusion increases urea reabsorption in the nephron collecting ducts [2,3]. This patient presented with elevated BUN levels, but with CRS1 progression, these levels escalated exponentially.

Bedside point-of-care ultrasound revealed a small pericardial effusion not present on initial TTE. The diagnosis in this case evolved from acute decompensated HF to a working diagnosis of pericardial constraint. The differential diagnosis for elevated, near-equalized diastolic pressures in this context included constrictive pericarditis, restrictive cardiomyopathy, and right ventricular (RV) infarction. Constrictive pericarditis was considered less likely given the acute onset and favorable response to anti-inflammatory therapy rather than a chronic fibrotic process. Restrictive cardiomyopathy was considered, given the patient's known diastolic dysfunction, though the acute clinical trajectory and new pericardial effusion argued against this as the primary mechanism. RV infarction was excluded by negative troponin and preserved RV function.

Uremic pericarditis was considered only after progression to severe azotemia (BUN >130 mg/dL), rather than the initial mildly elevated BUN. We acknowledge that diuresis is contraindicated in tamponade management, though the administration of furosemide on day 4 was a consequence of the diagnostic overlap between CRS1 and evolving pericarditis. While obstructive shock from cardiac tamponade was a component of our differential, effusion size and absent right atrium/RV diastolic collapse on TTE were not initially supportive. We acknowledge that near-equalization of diastolic pressures can occur in pure volume overload. In this case, elevated pulmonary capillary wedge pressure and preserved cardiac output argue against classic low-output tamponade physiology. Therefore, we interpret these findings as reflecting pericardial constraint superimposed on elevated filling pressures rather than definitive tamponade shock. The emergence of a new effusion, pleuritic pain, and the rise in BUN to 132 mg/dL support an inflammatory pericardial process contributing to impaired diastolic filling.

Tamponade physiology with a small effusion is uncommon, but it is possible with rapid fluid accumulation [4]. After a corrected clinical interpretation, anti-inflammatory therapy was initiated, which reduced pericardial inflammation and improved diastolic compliance rather than providing acute mechanical relief of pressure. Cardiac tamponade is a rare complication of uremic pericarditis in nondialyzed patients compared with HD-dependent end-stage renal disease patients (6% vs. 20% incidence, respectively) [5,6]. Platelet dysfunction due to severe uremia, along with apixaban, raised concern for a hemorrhagic effusion, prompting anticoagulation cessation [7,8].

Per the 2015 European Society of Cardiology (ESC) guidelines for pericardial disease, HD is a class IIa recommendation for treatment of pericarditis in renal failure [9]. However, our patient could not undergo HD due to severe hypotension and a lack of CRRT. In HD-refractory cases, pericardial drainage is a class IIb recommendation; however, given the small effusion size and high procedural risk for myocardial injury, pericardiocentesis was deferred. Per guidelines, nonsteroidal anti-inflammatory drugs and corticosteroids are a class IIb recommendation when HD is ineffective. Despite limited evidence in this scenario, our anti-inflammatory approach consisted of renally dosed colchicine and low-dose prednisone. The COlchicine for acute PEricarditis trial demonstrated the benefit of colchicine in idiopathic and viral pericarditis; however, its applicability to uremic pericarditis is uncertain. Despite limited direct evidence in this population, colchicine was used cautiously as adjunctive therapy [10].

## Conclusions

This case highlights that tamponade physiology exists on a spectrum of pericardial constraint. The absence of right ventricular collapse does not exclude pericardial constraint if the baseline intracardiac pressures are elevated. Clinicians should prioritize the clinical narrative, specifically the paradoxical response to diuresis, over static imaging criteria in patients with complex cardiorenal disease. Invasive hemodynamics must be interpreted cautiously, particularly in patients with concurrent HF and elevated filling pressures. Management should be individualized when standard therapies like HD are not feasible. In situations where mechanical drainage is high-risk, anti-inflammatory therapy may improve the clinical state by restoring pericardial compliance. While this approach was successful here, it should be viewed as an adjunct for inflammatory constraint rather than a replacement for decompression in high-pressure, classic cardiac tamponade.
